# Differences in the strength of cortical and brainstem inputs to SSA and non-SSA neurons in the inferior colliculus

**DOI:** 10.1038/srep10383

**Published:** 2015-05-20

**Authors:** Yaneri A. Ayala, Adanna Udeh, Kelsey Dutta, Deborah Bishop, Manuel S. Malmierca, Douglas L. Oliver

**Affiliations:** 1 Auditory Neurophysiology Laboratory. Institute of Neuroscience of Castilla Y León, University of Salamanca, C/Pintor Fernando Gallego, 1, 37007 Salamanca, Spain; 2 Department of Neuroscience, University of Connecticut Health Center, Farmington, CT 06030-3401, USA; 3 Department of Cell Biology and Pathology, Faculty of Medicine, University of Salamanca, Campus Miguel de Unamuno, 37007 Salamanca, Spain

## Abstract

In an ever changing auditory scene, change detection is an ongoing task performed by the auditory brain. Neurons in the midbrain and auditory cortex that exhibit stimulus-specific adaptation (SSA) may contribute to this process. Those neurons adapt to frequent sounds while retaining their excitability to rare sounds. Here, we test whether neurons exhibiting SSA and those without are part of the same networks in the inferior colliculus (IC). We recorded the responses to frequent and rare sounds and then marked the sites of these neurons with a retrograde tracer to correlate the source of projections with the physiological response. SSA neurons were confined to the non-lemniscal subdivisions and exhibited broad receptive fields, while the non-SSA were confined to the central nucleus and displayed narrow receptive fields. SSA neurons receive strong inputs from auditory cortical areas and very poor or even absent projections from the brainstem nuclei. On the contrary, the major sources of inputs to the neurons that lacked SSA were from the brainstem nuclei. These findings demonstrate that auditory cortical inputs are biased in favor of IC synaptic domains that are populated by SSA neurons enabling them to compare top-down signals with incoming sensory information from lower areas.

Animals, including humans are immersed in an ever-changing auditory scene. In order to detect unexpected events, the responses of some auditory neurons adapts to frequent sounds while they retain their excitability to rare sounds^1^. This neuronal phenomenon is called stimulus-specific adaptation (SSA) and has been thought to enhance the response to infrequent sounds and to reduce acoustic information redundancy^2^. SSA may contribute to evoked potentials related to deviance detection^3^,^4^,^5^ and to focus attention on the changes in the incoming stream of sensory information^2^. SSA responses are apparent in many neurons of the primary auditory cortex (Au1)^1^, medial geniculate body^6^,^7^, and inferior colliculus (IC)^8^,^9^,^10^.

The IC may be a site of dynamic control for the flow of biologically important acoustic information since it is a center for convergence of both ascending and descending auditory and non-auditory information^11^. The lemniscal pathway emerges from the central nucleus of the IC and projects to the core auditory cortex via the ventral division of the medial geniculate body. The non-lemniscal pathways originate from the cortex of the IC and the lateral tegmental system to project to the belt auditory cortex via the dorsal division of the medial geniculate body^12^,^13^. Interestingly, the non-lemniscal pathways in the midbrain have stronger inputs from the neocortex than those of the central nucleus of the IC^14^,^15^. Previous studies suggest that neurons with SSA are primarily located in the non-lemniscal subdivisions of the IC^9^,^16^,^17^.

Although the neurophysiology of SSA has been studied in great deal at the single neuron level in the IC^9^,^16^,^17^,^18^, rigorous attempts to correlate the anatomy of the auditory inputs and the physiology of SSA neurons are lacking. Here, we test whether neurons exhibiting SSA and those without are part of the same networks in the IC. We studied neurons exhibiting or lacking SSA in the IC and then marked the sites of these neurons with a retrograde tracer to correlate the source of the inputs with the physiological response. This approach is designed to detangle the connectivity of the so-called network for auditory deviance detection^4^.

## Results

**Figure 1:**
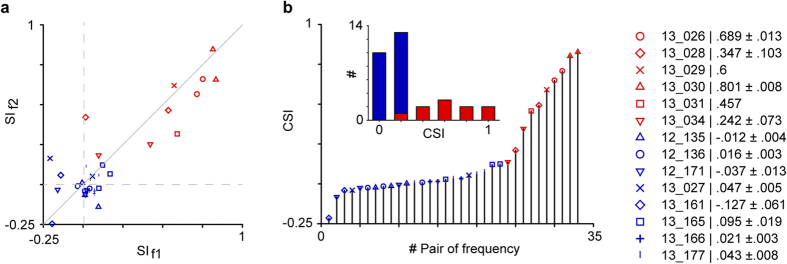
SSA indices of the response of neurons that exhibit SSA (red) and of those non-SSA neurons that lack SSA (blue). From one to four pairs of frequencies were presented for each neuron (symbols). (**a**) The Frequency-Specific Index (SI) was similar for both frequencies (f1, f2) presented under the oddball paradigm resulting in SI values aligned along the diagonal line (Wilcoxon Signed Rank Test, p = 0.08). (**b**) The non-SSA neurons had Common-SSA Index (CSI) values lower than 0.18 for all the pair of frequency tested. The SSA neurons had CSI values higher than 0.18 for most of the pair of frequency tested (9/10). The CSI = 0.18 was used as cutoff value to separate SSA from non-SSA neurons. The differential distribution of the CSI values for the SSA and non-SSA group is shown in the inset. The right inset showed the neuron number and the mean ± s.e.m. CSI value for each neuron when two or more frequencies were presented.

Since the main goal of this study was to determine the inputs to regions of IC that contain neurons that either exhibit significant SSA or lack SSA, first we recorded the extracellular single-unit responses to the oddball paradigm from 14 IC neurons (one per animal) and then made a minute injection of FG at the recording site. Two to four pairs of frequencies were used in the majority of neurons (12/14), and these elicited SI values ranging from −0.246 to 0.846 (Fig. 1a) and CSI values ranging from −0.214 to 0.827 (Fig. 1b). Neurons were classified as showing SSA when the CSI value exceeded 0.18 as in previous studies^7^,^16^,^17^. Thus, six neurons in our sample exhibited significant SSA levels (SSA cases), while eight neurons responded similarly to the deviant and standard tones across trials and, therefore, lacked SSA. The latter non-SSA neurons were used for comparison. Importantly, these two groups of neurons are easily distinguishable based solely on the presence or absence of SSA regardless of other response properties. The sizes of the FG injections were similar across the cases as well as the quality of the retrograde labeling of the neurons.

In the following results, we will first present the physiology and anatomy of two SSA cases; one with a low frequency BF and the other with a high frequency BF that are typical and representative of the whole sample. Then, we will compare these to the responses and inputs to the recording sites of two other neurons that lack SSA and that also showed low- and high-frequency BFs.

### SSA Cases

**Figure 2:**
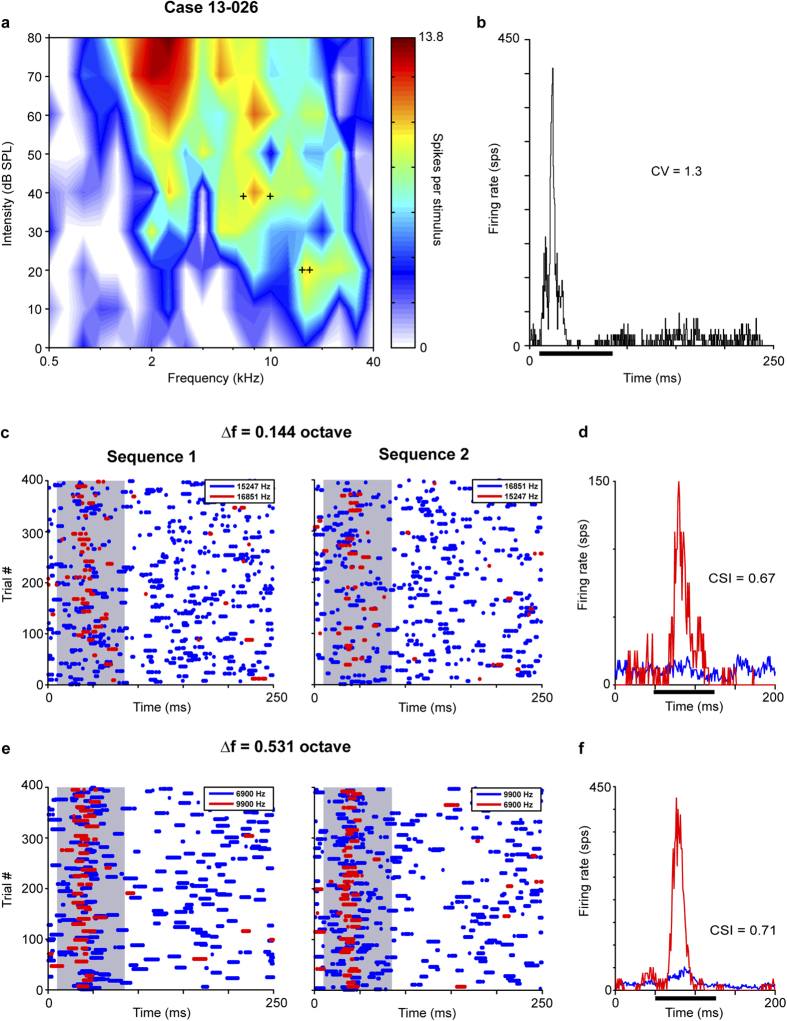
Low-frequency neuron with SSA. The adaptation to the standard tone was reflected by positive SSA index in this low-frequency SSA neuron. (**a**) Broadly-tuned frequency response area with a characteristic frequency of 2.52 kHz. (**b**) Transient and irregular firing pattern to the characteristic frequency. Dot rasters (**c**,**e**) and normalized PSTH (**d**,**f**) for two pairs of tested frequencies with separations of 0.144 and 0.531 octave. The pairs of frequencies are indicated within the frequency response area (+). The PSTHs (**d**,**f**) represent the mean response from both oddball sequences (1 and 2) showing a higher neuronal response to the deviant tone (red) than to the standard (blue). Case 13-026. PSTH bin size = 1 ms. CV, coefficient of variation; CSI, common SSA index. sps, spikes per second. Tone duration represented by bar and shaded areas.

We show two representative neurons from the six cases with significant SSA levels. The first neuron had a low BF at 2.52 kHz at 80 dB SPL and a complex FRA tuned to a wide spectrum of frequencies (Fig. 2a). Its broad bandwidth was similar at different intensity levels (Q_10_ = 0.51, Q_30_ = 0.56). The neuron showed a transient response and an irregular firing pattern at BF (CV = 1.27, Fig. 2b). Sensitivity to SSA was demonstrated by the adaptation of firing in response to the standard tone. This resulted in a CSI = 0.67 for a closely spaced pair of frequencies (Δf = 0.144 octaves, Fig. 2c,d). When the frequency contrast increased to Δf = 0.531 octaves, the CSI also increased to 0.71 (Fig. 2e,f). The latency of the neuronal response to the deviant stimulus was shorter (FSL = 26.568 ms ± 10.07, red) than to the standard one (FSL = 30.92 ms ± 12.52, blue) for both frequency contrast (Mann-Whitney Rank Sum Test, p = 0.016).

**Figure 3:**
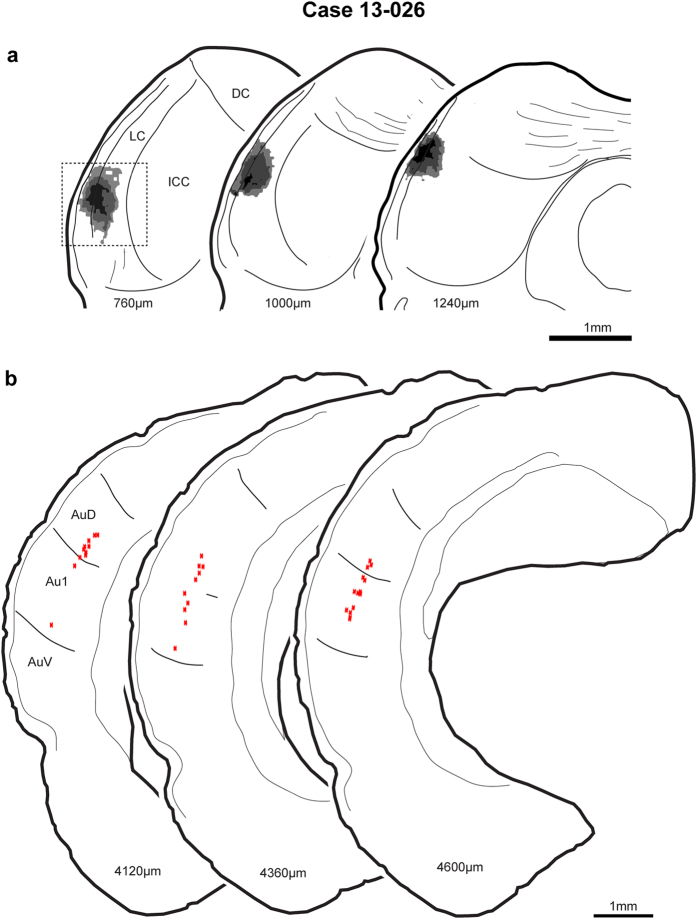
Injection site of SSA neuron and retrograde labelling for case 13-026. The injection site for the high-frequency SSA neuron was confined to the lateral cortex of the IC (**a**) and labeled neurons 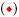 were only found in auditory cortices including primary and non-primary areas (**b**). Section distance is relative to caudal-most section of IC. LC, lateral cortex of IC; DC, dorsal cortex of IC; ICC, central nucleus of IC; Au1, primary auditory neocortex; AuD, dorsal auditory area; AuV, ventral auditory area.

**Figure 4:**
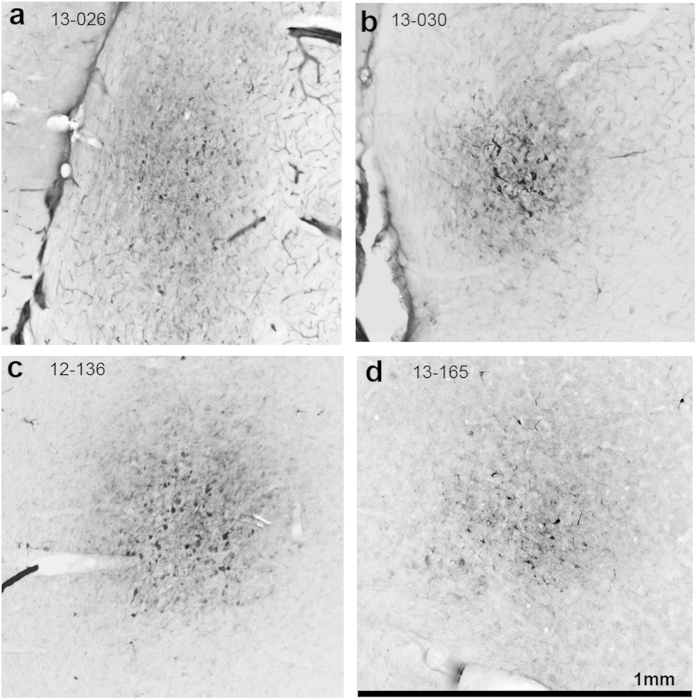
Photomicrographs showing the injection site for SSA cases 13-026 (**a**) and 13-030 (**b**) and non SSA cases 12-136 (**c**) and 13-165 (**d**). Each photomicrograph showing the injection site is identified with a box in the illustration of each case (cf. Figs. 3, 6, 8, and 10).

In this case, the injection of the region containing the recorded neuron resulted in extensive retrograde labeling in the auditory cortex but little labeling in the brainstem. The FG injection was confined to the lateral cortex with a 350 μm-wide center (Fig. 3a, section 760, black; the box indicates the photomicrograph illustrated in Fig. 4a), and it followed the dorsal to ventral electrode track. This orientation matches that of the recording pipette which was tilted 20° in a rostro-caudal direction and advanced from dorsal to ventral. It was primarily confined to the small-cell middle layer and the deepest layer of large cells^19^. No brainstem labelling was found, but heavily labeled neurons were found through the auditory cortices including areas Au1 and AuD (Fig. 3b). Those cortical neurons were mostly found at a similar depth of about 700−880 μm and displayed the morphology of layer 5 pyramidal cells. In all cases, there was labeling in the ICC apart from the injection site that we interpret as retrograde labeling.

**Figure 5:**
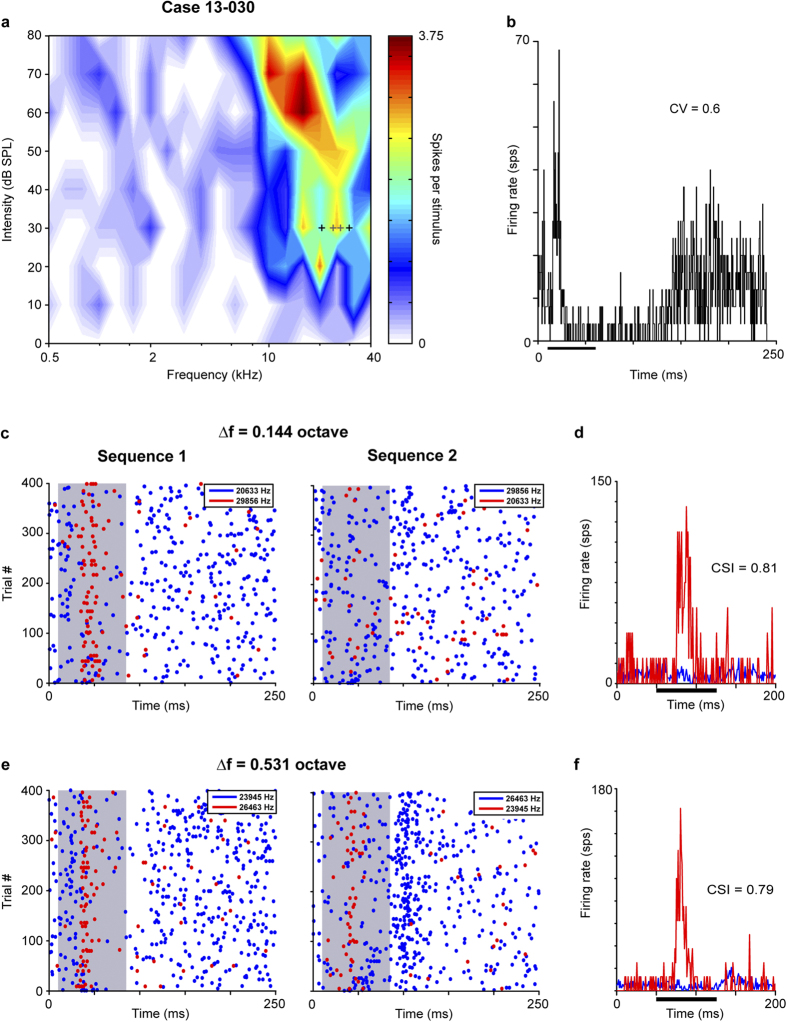
High-frequency neuron with SSA. The significant SSA level exhibited by this neuron was reflected by the stronger response to the deviant tone and the faint response to the standard tone. (**a**) Frequency response area tuned to high frequencies with a low threshold. (**b**) The irregular response and spontaneous discharge to best frequency (15.853 kHz) was evident in the PSTH. Dot rasters (**c**,**e**) and normalized PSTH (**d**,**f**) for two pairs of tested frequencies as deviant (red) and standard (blue) with separations of 0.144 and 0.531 octave. Case 13-030. Abbreviations as in Fig. 2.

The second typical case also had a neuron exhibiting strong SSA. The neuron had a broad frequency response area (Q_10_ = 1.03, Q_30_ = 0 1.02) and was tuned to high frequencies around 10 kHz and above. It showed a non-monotonic rate level response to both the BF (15.853 kHz, MI = 0.53) and CF (31.915 kHz, MI = 0.6) (Fig. 5a). The response to the BF at 60 dB SPL was transient and irregular (CV = 0.62) with a suppression in the spontaneous activity that lasted more than 100 ms (Fig. 5b). In terms of SSA, the neuron´s differential response to the deviant and to the standard tone produced a high CSI value for pairs of frequencies separated by 0.144 octave (CSI = 0.81) (Fig. 5c,d) and 0.531octave (CSI = 0.79) (Fig. 5e,f).

**Figure 6:**
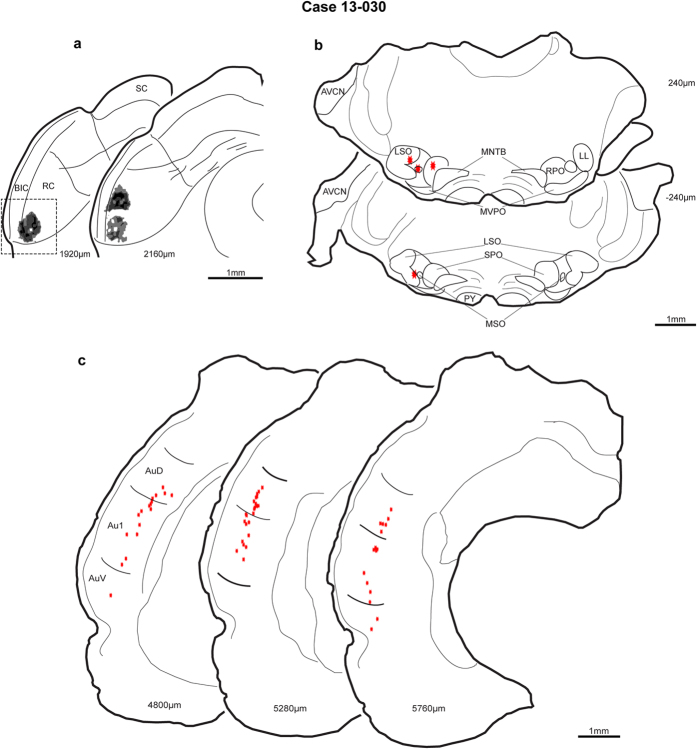
Injection site and retrograde labelling for case 13-030. (**a**) The injection site in the ventral lateral and rostral cortex of IC. Rostrally, two tracer deposits appeared. (**b**) Sparse brainstem labelling 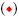. (**c**) Numerous labeled neurons throughout the auditory cortex. SC, superior colliculus; BIC, brachium inferior colliculus; RC, rostral cortex of the IC; AVCN, anteroventral cochlear nucleus; LSO, lateral superior olive; MNTB, medial nucleus of the trapezoid body; LL, lateral lemniscus; MVPO, medioventral periolivary nucleus; SPO, superior periolivary nucleus; MSO, medial superior olive; RPO, rostral periolivary region; PY, pyramidal tract. Other abbreviations as in Fig. 3.

The site of this neuron received extensive inputs from the neocortex but only sparse input from the superior olive. The injection site was rostral and lateral in the cortex of the IC with a center of 280 μm at its maximum extent (Fig. 6a, section 1920 μm, black; the box indicates the photomicrograph illustrated in Fig. 4b). In the most rostral sections (Fig. 6a, 2160 μm), two tracer deposits were found but the upper one had the heaviest labelling. Very few FG-labeled somata were located in the brainstem. Single labeled neurons were found in the superior periolivary nucleus (SPO), medial superior olive and medial region of the lateral superior olive (Fig. 6b). The location of the labeled neurons in the medial superior olive and lateral superior olive indicated that the retrograde labelling pattern corresponded well with the high frequency tuning of the IC recorded neuron. On the other hand, the neocortical labelling pattern was similar and more abundant than the previous SSA case (Fig. 3). Retrogradely labelled cortical neurons extended to very rostral sections (5760 μm) and were distributed throughout the dorsoventral axis in areas AuV, Au1 and AuD. The highest density of labeled neurons were those clustered in AuD area of auditory cortex (Fig. 6c).

### Non-SSA Cases

**Figure 7:**
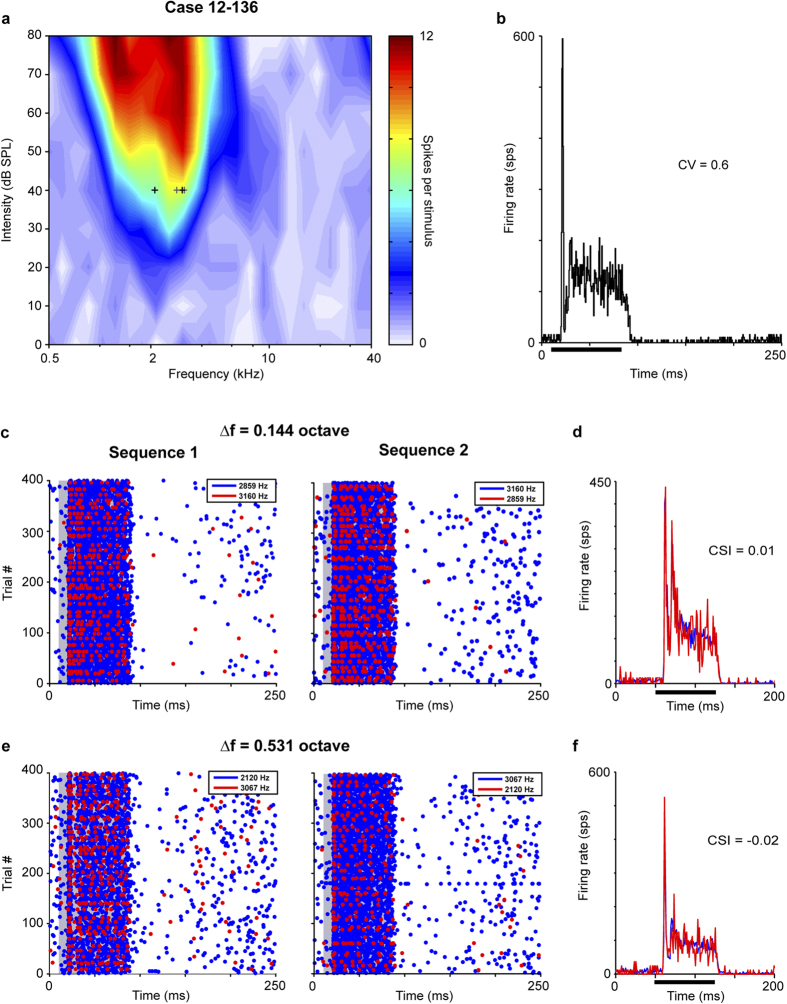
Low-frequency neuron without SSA. Typical V-shaped frequency response area and lack of SSA in a low-frequency neuron. (**a**) This neuron is tuned to low frequencies (best frequency and characteristic frequency = 2.61 kHz). (**b**) On-sustained response to the best frequency. Lack of SSA in sequence 1 and 2 with 0.114 octave separation shown in raster (**c**; standard, blue; deviant, red) and normalized PSTH (**d**). Similar lack of SSA with 0.531 octave separation (**e**,**f**). Case 12-136. Abbreviations as in Fig. 2.

Neurons that lack SSA were used to compare both physiological and anatomical data. The first example neuron had a low BF with a narrow V-shaped FRA (Fig. 7a), and a monotonic rate intensity function (MI = 1). The temporal response to the BF (2.61 kHz) showed an on-sustained response type (Fig. 7b). In this neuron, we stimulated with two pairs of frequencies, one with a ∆f of 0.144 octave (Fig. 7c,d), and the other with a ∆f of 0.531 octave (Fig. 7e–f). The neuronal responses to the deviant and standard tone presentations during the two oddball sequences are illustrated by dot rasters (Fig. 7c–e) and the corresponding mean peri-stimulus time histogram (PSTH, Fig. 7d,f). This neuron did not show SSA since its response strength and pattern to the deviant and standard tone was very similar regardless of the frequency separation between tones. This lack of adaptation in the response was reflected by its very low CSI value, 0.01 and –0.025 for ∆f = 0.144 and 0.531, respectively.

**Figure 8:**
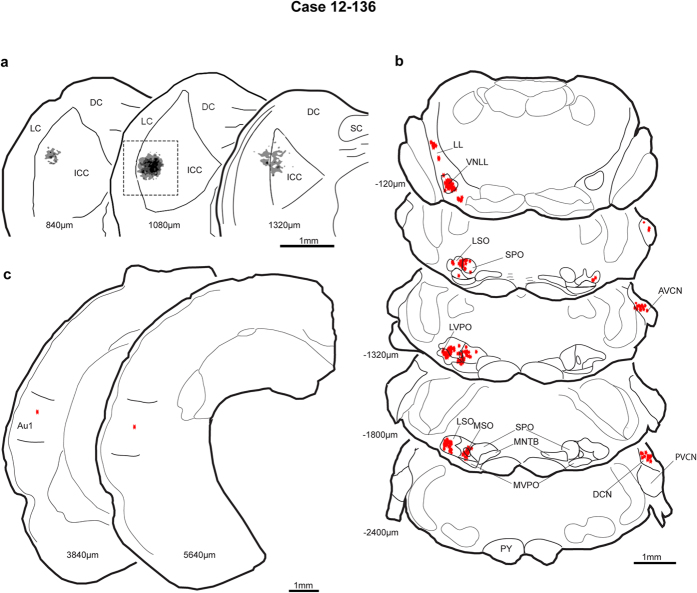
Central nucleus injection site and retrograde labelling of the brainstem and auditory cortices of the non-SSA case 12-136 (**a**) Injection site in IC in rostral central nucleus and adjacent lateral cortex. (**b**) Numerous labeled neurons 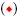 in brainstem but sparse label in the auditory cortices (**c**). Case 12-136. DCN, dorsal cochlear nucleus; LVPO, lateroventral periolivary nucleus; PVCN, posterioventral cochlear nucleus; VNLL, ventral nucleus of lateral lemniscus. Other abbreviations as in Figs. 3 and 6.

In this case, the small region around the neuron received extensive brainstem inputs. The center of the injection site was rostral and lateral in the central nucleus of the IC and reached a maximum diameter of 450 μm (Fig. 8a; the box on section 1080 μm indicates the photomicrograph illustrated in Fig. 4c). The injection also extended into the adjacent lateral cortex of IC (Fig. 8a). The resulting retrograde labelling in the brainstem nuclei was typical with many labeled neurons in the ipsilateral superior olivary complex, ipsilateral ventral nucleus of the lateral lemniscus (LL), and contralateral cochlear nucleus (CN) (Fig. 8b). Labeling was prominent in the ipsilateral medial and lateral superior olives and ipsilateral SPO. Little labeling was seen in the contralateral superior olivary complex. Labeling in the nuclei of the LL was concentrated ventrally in the ventral nucleus and very little in the intermediate and dorsal nuclei of the LL. Dense clusters of labeled neurons were found in the contralateral dorsal CN and anteroventral CN (Fig. 8b). In contrast to the robust labelling displayed by the brainstem nuclei, only two labeled neurons were found in the neocortex, and these were located in Au1 (Fig. 8c).

**Figure 9:**
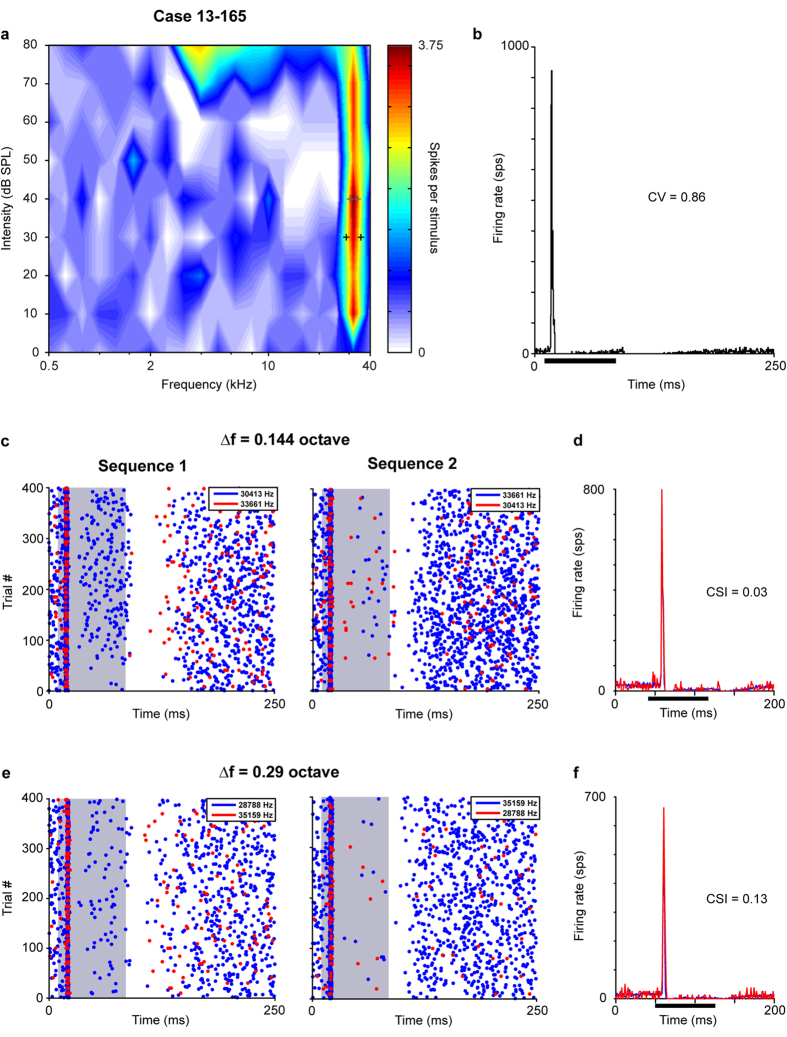
High-frequency neuron without SSA. This neuron had a very narrow frequency response area (**a**) and an onset response pattern to its characteristic frequency (**b**). The neuron did not show SSA in its response to the oddball paradigm for two pairs of frequencies (standard, blue; deviant, red) with different separation; 0.144 octave (**c**,**d**) and 0.29 octave (**e**,**f**). Case 13-165. Abbreviations as in Fig. 2.

The second non-SSA neuron had a high BF, a narrow FRA (2.36 and 2.75 for the Q_10_ and Q_30_, respectively) flanked by an inhibitory area at the low frequency region, and a non-monotonic rate intensity function (MI = 0.66) (Fig. 9a). At the BF of 32.13 kHz (at 40 dB SPL), this neuron had an onset response with a short first spike latency (7.495 ms ± 0.5861) across trials (Fig. 9b). Despite the difference in the BF, FRA, rate level function, and temporal patterns compared to the first non-SSA neuron, the strength and timing of the response to the deviant and standard stimulus was still very similar as observed in the rasters (Fig. 9c,e) and PSTHs (Fig. 9d,f) obtained from two pair of tested frequencies. The CSIs ranged from 0.03 for 0.144 octave separation to 0.13 for ∆f = 0.29 octave.

**Figure 10:**
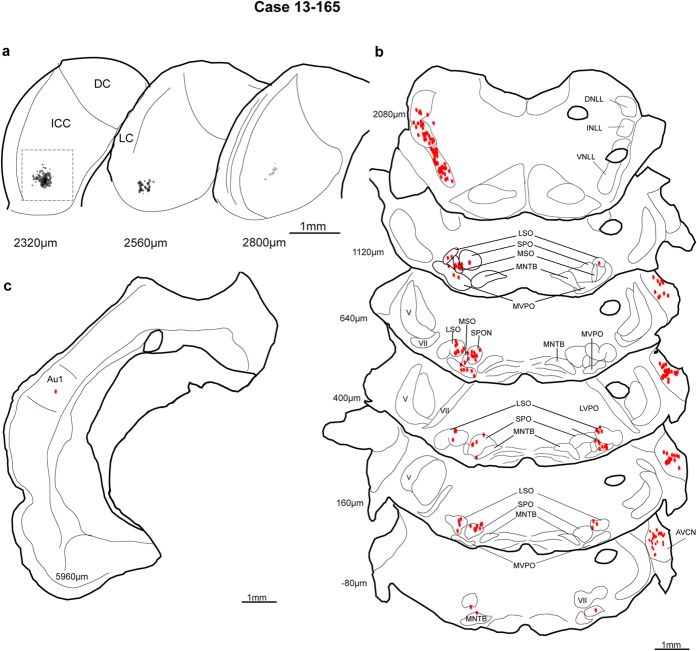
IC injection site and retrograde labelling for case 13-165. (**a**) Injection site in the ventromedial central nucleus. (**b**) Strong labelling in the brainstem sections corresponding to the high frequencies areas of the superior olivary complex and nucleus of the trapezoid body. (**c**) Weak auditory cortex labelling. DNLL, dorsal nucleus of lateral lemniscus; INLL, intermediate nucleus of lateral lemniscus. V, trigeminal tract; VII, facial nerve. Other abbreviations as in Figs. 3, 6, 8.

Similar to the first non-SSA case, most inputs to the region of the neuron were in the brainstem. As predicted from the high BF, the recording site was located in the ventral central nucleus and reached a maximum diameter of 350 μm (Fig. 10a; the box on section 2320 μm indicates the photomicrograph illustrated in Fig. 4c). The most prominent labelling was throughout the ventral nucleus of the LL and SPO (Fig. 10b). As expected from the high BF in the recording site, the medial, high frequency lateral superior olive was retrogradely labeled bilaterally and labeled neurons were found in the middle and dorsal anteroventral CN. A tonotopic organization for the modest labeling in the periolivary nuclei of superior olivary complex was less obvious. At the level of the midbrain, the three ipsilateral nuclei of the LL (dorsal, intermediate, and ventral) contained labeled neurons as did the contralateral dorsal and intermediate LL (not shown). Scattered labelling was also found in the central nucleus and lateral cortex at the level of the injection site. As in the previous non-SSA case, the cortical labelling was very modest with only one labeled neuron located in Au1 (Fig. 10c).

### Characteristics of All Sampled Neurons

**Figure 11:**
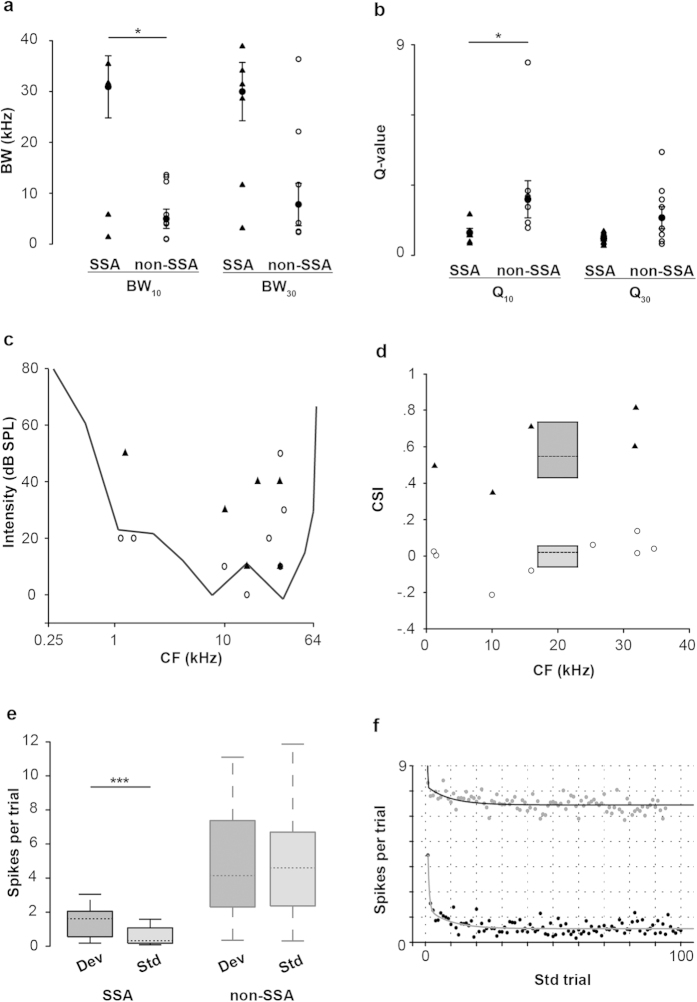
SSA and non-SSA neurons differed in frequency response areas, deviant sensitivity and response strength, but not in threshold. (**a**) Distribution and median ± s.e.m. of bandwidth (BW) of SSA and non-SSA neurons at 10 and 30 dB SPL above threshold. (**b**) Distribution and median ± s.e.m. of Q values at 10 dB and 30 dB SPL above threshold. SSA neurons had broader frequency tuning at 10 dB SPL above threshold (Wilcoxon Signed Rank Test, * p < 0.05). (**c**) Distribution of the characteristic frequencies (CF) for SSA (▲) and non-SSA (○) neurons relative to rat audiogram. (**d**) Box plot of the SSA indices (CSI) of the SSA (▲) and non-SSA (○) groups (median value, dashed lines). (**e**) Box plots of the population spike rates for SSA and non-SSA neurons to deviant (Dev) and standard (Std) stimuli. The dashed lines within each box represent the median values, the edges of the box delimit the 25^th^ and 75^th^ percentiles and the whiskers bars indicate the 10^th^ and 90^th^ percentiles. The firing response to the deviant and standard tone only differs in the SSA group (Wilcoxon Signed Rank Test, *** p < 0.001). (**f**) Fit of the population response to the standard tone for SSA (•) and non-SSA (•[grey]) neurons to a double exponential function. There is a rapid and pronounce decay from the first standard trial in the response of SSA neurons but not in non-SSA neurons.

A clear difference between neurons that exhibit SSA and those neurons without SSA was the bandwidth of their response areas. SSA neurons were characterized by their broadband tuning, whilst most non-SSA neurons had V-shaped FRAs (n = 5) according to the receptive field classification previously suggested^20^,^21^. The remaining non-SSA neurons had V-shaped FRAs but non-monotonic at CF (n = 1) and narrow response areas (n = 2). The differential spectral sensitivity between both groups was reflected in the population BW and Q-values (Fig. 11a, b). At 10 dB SPL above threshold, the SSA neurons already had a bandwidth of 30.889 ± 14.929 kHz that remained similar at higher intensities (29.952 ± 14.017 kHz) (Paired t-test, t = −1.546, p = 0.183).

SSA and non-SSA groups did not differ in their monotonicity (MI = 1 ± 0.136, 1 ± 0.18 for non-SSA and SSA group, respectively), firing regularity (CV = 0.75 ± 0.295, 1.271 ± 0.652), frequency tuning (CF = 20.67 ± 13.87, 17.993 ± 12.104 kHz) or thresholds (20 ± 15.119, 35 ± 16.733 dB SPL) (For all parameters, Mann-Whitney Rank Sum Test, p > 0.05). Sample of SSA and non-SSA neurons included both the low and high extremes of the rat audiogram. Moreover, the neuronal thresholds (Fig. 11c) were within, or very close to, the average limens of the audiogram^22^. There was no correlation between the CF and the CSI for either SSA (Spearman Rank Order Correlation Coefficient = 0.242, p > 0.05) or non-SSA neurons (Coefficient = 0.182, p > 0.05) (Fig. 11d).

Obviously, there was a significant difference in the amount of SSA between SSA (CSI = 0.546 ± 0.172) and non-SSA groups (CSI = 0.0195 ± 0.102) (t-test, t = −7.744, P < 0.001). Since the majority of the recorded neurons exhibited spontaneous discharge (7.7656 ± 5.3142 spikes per second, n = 13 neurons) and this affects the CSI measurement^23^, we recalculated the CSI by subtracting the mean spontaneous discharge from the responses to the deviant and standard tones. After subtraction, the median population CSI values changed slightly (SSA, CSI = 0.576 ± 0.205; non-SSA, CSI = −0.00491 ± 0.141). Consistent with the population CSI, the median spike count was higher in response to the deviant tone (1.625 ± 1.5963) than to the standard tone (0.3222 ± 0.5141) for the SSA group (Wilcoxon Signed Rank Test, Z = −4.107, p < 0.001) (Fig. 11e); while the response to both tones was similar in the non-SSA group (4.15 ± 2.987 and 4.5972 ± 3.0487 for deviant and standard stimulus, respectively) (Wilcoxon Signed Rank Test, Z = −0,464, p = 0.646). The temporal dynamics of the response of the SSA neurons to the standard tone (Fig. 11f, bottom plot) followed a double exponential function (r^2^ = 0.6754, SSE: 5.048) indicating that the strength of the response rapidly decays during the first presentations of the standard tones as described previously^24^. On the contrary, the response of the non-SSA neurons to the standard tone (Fig. 11f, upper plot) did not suffered an strong decay reflected by a poor fit to the same function (r^2^ = 0.2613, SSE: 6.54).

In all cases, there was a good correlation between the neuronal response properties and the morphology. The SSA neurons were localized in the lateral (n = 3) and rostral cortex of IC (n = 3). On the other hand, the sites of the non-SSA neurons were confined to the dorsolateral (n = 2), ventrolateral (n = 3), middle (n = 2) and very rostral (n = 1) regions of the central nucleus.

## Discussion

We show a segregation of cortical and brainstem inputs to the sites where SSA and non-SSA neurons are located in the IC. Consistent with previous studies, SSA neurons were located in the cortex of the IC, whilst the IC neurons that do not exhibit SSA were confined to the central nucleus of the IC^9^,^16^,^17^. The injection sites in the SSA cases received heavy inputs from Au1 as well as from areas dorsal and ventral to Au1, while the non-SSA cases were strongly innervated by brainstem inputs. Since these results show that regions in IC containing neurons with SSA have a pattern of inputs that differs from regions without SSA, it suggests a unique network organization to generate SSA in the IC.

### Sources of inputs to regions with and without SSA

All SSA cases showed a consistent pattern of afferent projections, *i.e*., strong inputs from the auditory cortex and very poor or even absent projections from the brainstem nuclei. Thus, one of the main sources of input to neurons exhibiting SSA in the IC is the auditory cortex. The distribution of the retrogradely labeled neurons in the cortex covered not only the Au1 area, but it also included other high-order auditory cortical areas that may be involved in deviance detection as suggested by recent studies^25^. In addition to the cortical input, the injection sites in the lateral cortex of IC invariably labeled neurons in ICC with retrograde transport. This may provide important auditory inputs to SSA neurons. However, with the methods used here it is difficult to separate ICC neurons labeled due to fibers of passage from those with direct inputs to SSA neurons. The projections from ICC to the LC were documented earlier with autoradiographic anterograde transport methods that are not subject to the fiber of passage artifact^26^. So, it is certain that collateral axons from ICC neurons may terminate in the LC in route to the brachium of the IC and the medial geniculate body^27^,^28^,^29^.

Neurons that lacked SSA differed markedly from SSA neurons in their source of inputs. The major sources of input to the non-SSA cases were from brainstem nuclei, *i.e*., CN, nuclei of the superior olivary complex and LL, consistent the notion that ICC neurons mainly integrate ascending excitatory and inhibitory information. The distribution of the labeled neurons followed the general tonotopic arrangement described for the ascending projections to the recordings sites within the central nucleus^30^,^31^. The weak cortical projections seen after non-SSA injections were distinct from those in the SSA cases. Furthermore, the few retrograde labeled neurons found in the auditory cortex were restricted to Au1. This is consistent with previous reports of weaker projections from auditory cortex to ICC than to LC or DC^14^,^15^.

The injections in this study were intentionally designed to produce local minute deposits of tracer and used micropipettes with tips that allowed good isolation of single units. Because of the small size of the injections, relatively small numbers of neurons were labeled in each case, and some could be missed. Not all of the neurons known to project to the ICC or LC were found in every case, e.g., neurons from the CN^32^,^33^, the medial superior olive^34^,^35^, the SPO^32^,^36^, the LL^37^, and the contralateral IC^28^,^38^.

The retrogradely labeled neurons identify the sources of projections to a small region in the vicinity of the recorded neuron consistent with a small functional zone. Despite the small size of the injection, the diffusion of the FG from the tip of the electrode undoubtedly extends beyond the dendritic field of the single neuron under study. Presumably, all the neurons in the small region around the neuron in the injection site share the same input sources. While this does not rule out exclusive projections to SSA or non-SSA neurons that are side by side, it is consistent with the notion that neurons with SSA are organized into functional zones at least as small as the injection sites. Synaptic domains, *i.e.*, small groups of neurons that share the same subset of synaptic inputs^39^,^40^,^41^ have been identified in ICC. In the present context, the consistent pattern of cortical labelling may reflect common projections to synaptic domains for SSA neurons in the cortex of IC. Those synaptic domains may account for other physiological properties exhibited by SSA neurons.

### Relationship of inputs to neuronal function

The injection sites confirmed that SSA neurons are confined to the non-lemniscal subdivisions of the IC, *i.e.,* lateral and rostral cortices^13^. A broad spectral sensitivity and a lower driven rate characterize the response properties of auditory neurons of the non-lemniscal pathway^13^,^42^. In contrast, the response features of non-SSA neurons match those described for the lemniscal auditory pathway^13^,^42^,^43^. The location and frequency tuning of the recorded neurons in ICC was well-matched to the tonotopic organization of the central nucleus^44^. The non-SSA neurons were very sharply tuned (Fig. 11a,b) and responded to the occurrence of the deviant and standard tones with a similar and constant response strength along all the stimulus presentations (Fig. 11e,f).

The pattern of connections described above is also consistent with the function of the corticocollicular projections on SSA responses in IC as demonstrated by the inactivation of the auditory cortex^45^. The corticofugal projection exert mainly gain control over the SSA response and affected both the response to the deviant and to the standard tone without abolishing the difference between them. The cortical inactivation elicited changes in the SSA index in either direction; increasing or decreasing the CSI. However, very few SSA responses were generated *de novo* or abolished completely by the inactivation of the cortical inputs. The diverse effects of cortical manipulation on SSA responses might be explained by cortical inputs to synaptic domains in IC that contain neurons with different SSA sensitivities. More likely, SSA domains might receive inputs from different areas of auditory cortex. Diverse cortical effects might be indirect since cholinergic neurons of the tegmental nucleus that project directly to IC also receive inputs from auditory cortex^46^.

### Functional significance

The theory of predictive coding relies in the comparison between the incoming bottom-up sensory input and the memory trace formation in top-down fashion^47^. Then, a higher order center of processing sends predictions to the level below, which reciprocate bottom-up signals. These signals are prediction errors that report discrepancies between top-down predictions and representations at each level^48^ without requiring attentional processing of the stimulus or cognitive control of the predictions^49^,^50^. While current studies of predictive coding consider mostly cortical processing^51^, here we proposed that SSA neurons in the IC participate in this processing through corticofugal projections^52^,^53^,^54^.

Our results support the notion that SSA neurons generate an error signal by comparing ascending and descending inputs. The present data show that SSA neurons are in a position in the auditory pathway (*i.e*., non-lemniscal subdivisions) to compare higher-level signals with incoming sensory information. We postulate that when the same stimulus evokes converging simultaneous excitatory inputs to the SSA neuron from the auditory cortex and the central nucleus of the IC, the SSA neuron adapts (as occurs in response to the common sound). However, when those inputs differ (as when a rare sound occurs), the SSA neuron fires generating an error signal. SSA neurons in the IC are in a suitable circuit to send the error signal to the neocortex by connections in the non-lemniscal pathways^55^ driving neuronal activity in the belt auditory cortex to adjust and update the sensory representation^56^. The present data suggests a fast, top-down adjustment of IC activity is made possible by the feedback loop from the cortex. This is consistent with the corticofugal modulation of IC neurons that effects a short-term plastic reorganization in the frequency domain^52^ and on SSA responses^45^. Thus, SSA neurons in the IC may be useful in the context of the continuous interaction between a top-down flow of sensory predictions and a bottom-up flow of the incoming sensory representation.

## Methods

### Subjects and surgical procedures

Experiments were performed on 14 female rats (*Rattus norvegicus*, Rj: Long-Evans) with body weights ranging 136–222 g. All surgery and recording procedures, tracer injections as well as perfusions were conducted at the University of Salamanca. The experimental protocols were approved by Animal Care Committees of the University of Salamanca and used methods conforming to the standards of the University of Salamanca Animal Care Committee and the European Union (Directive 2010/63/EU) for the use of animals in neuroscience research. Detailed procedures are given elsewhere^9^,^21^,^57^. Anesthesia was induced using a mixture of ketamine chlorohydrate (30 mg/kg, I.M., Imalgene 1000, Rhone Méreuse, Lyon, France) and xylazine chlorohydrate (5 mg/ Kg, Rompun, Bayer, Leverkusen, Germany). The animal was placed inside a sound-attenuated room in a stereotaxic frame in which the ear bars were replaced by a hollow speculum that accommodated a sound delivery system. Atropine sulphate (0.05 mg/kg, s.c., Braun, Barcelona, Spain) was administered to reduce bronchial secretions. During surgery and recording, the body temperature was monitored with a rectal probe and maintained at 38 °C with a thermostatically controlled electric blanket. A craniotomy was made in the caudal part of the left parietal bone to expose the cerebral cortex in order to gain access to the left IC.

### Electrophysiological recordings and acoustical stimuli

Extracellular responses of a well-isolated neuron were recorded in the left IC of each animal with a glass micropipette (tip OD=4 μm, 3.5–5.5 MΩ) that was used for both recording and iontophoretic injections. Pipettes were filled with the 2% Fluorogold (FG, Fluorochrome, Denver, CO, USA) in a sterile saline solution (0.9% NaCl). The IC was approached from 20° relative to the frontal plane so that the electrode moved caudal and ventral during the penetration. The electrode was lowered into the brain with a piezoelectric microdrive (Burleigh 6000 ULN) mounted on a stereotaxic manipulator to a depth between 3.5–5 mm where acoustically driven responses were found.

Search stimuli were pure tones or noise bursts delivered through a sealed acoustic system^43^ using two electrostatic loudspeakers (TDT-EC1) driven by a TDT System 2 (TDT, Tucker-Davis Technologies, Florida, USA) that was controlled by custom software for stimulus generation and on-line data visualization. Action potentials were recorded with a TDT BIOAMP amplifier, the ×10 output of which was further amplified and bandpass-filtered (TDT PC1; fc: 0.5–3 kHz) before passing through a spike discriminator (TDT SD1). Spike times were logged at one microsecond resolution on a computer by feeding the output of the spike discriminator into an event timer (TDT ET1) synchronized to a timing generator (TDT TG6). Spike times were displayed as dot rasters sorted by the acoustic parameter varied during testing.

After a neuron was isolated, pure tone stimuli (75 ms with a 5 ms rise/fall time) were delivered to the ear contralateral to the recording site and the monaural frequency response area (FRA) was obtained. Specifically, the combination of frequencies and intensities capable of evoking a response, was obtained with an automated procedure consisting of 5 stimulus repetitions at each frequency (from 0.5 to 40 kHz, in 20–25 logarithmic steps) and intensity step (steps of 10 dB, from 0 to 80 dB SPL) presented randomly at a repetition rate of 4 Hz.

### Stimulus presentation paradigm

Monaural stimuli to the contralateral ear were presented in an oddball paradigm similar to that used to record mismatch negativity responses in human studies^58^ and more recently in animal studies of SSA in the auditory cortex^1^ and auditory midbrain^6^,^7^,^9^. Briefly, this paradigm consists in a flip-flop design of two pure tones at two different frequencies (f1 and f2) that elicited a similar firing rate and response pattern at the same level of 10–40 dB SPL above threshold. Both frequencies were within the excitatory response area of the neuron. A train of 400 stimulus presentations containing both frequencies was delivered in two different sequences (sequence 1 and 2). The repetition rate of the train of stimuli was 4 Hz, as it has been previously demonstrated to be suitable to elicit SSA in IC neurons of the rat^9^,^10^. In sequence 1, the f1 frequency was presented as the standard (*i.e.*, high probability within the sequence: 90%); interspersed randomly among the standards were the f2 frequency deviant stimulus (*i.e.*, low probability: 10%, respectively). After obtaining one data set, the relative probabilities of the two stimuli were reversed, with f2 as the standard and f1 as the deviant in the sequence 2. These two sequences are a flip-flop design since the identity of the standard and deviant are reversed. The responses to the standard and deviant stimuli were normalized to spikes per stimulus, to account for the different number of presentations in each condition, because of the different probabilities. The frequency separations (Δf) between f1 and f2 varied between 0.14 octaves to 0.53 octaves since the frequency pairs were chosen to evoke similar firing rates in responses to both tones.

Once the electrophysiological recording was completed, FG was iontophoretically ejected from the recording pipette into the recording site with 0.5 μA current applied for 1 s to produce a small tracer deposit. Seven days after the surgery, the animals were deeply anesthetized with sodium pentobarbital (60 mg/kg, Dolethal, Vétoquinol, Madrid, Spain) and perfused transcardially with Ringer´s solution and 4% paraformaldehyde in 0.1 M phosphate buffer (PB, pH 7.4).

### Analysis of neuronal responses

For each neuron, the amount of SSA was quantified by the Common-SSA Index (CSI) and the Frequency-Specific SSA Index (SI) used previously^1^,^7^,^9^,^24^. The CSI and SI reflect the normalized difference between the neuronal response to the deviant stimulus and the response to the standard one. The CSI is defined as


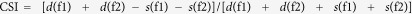


where d(f) and s(f) are responses to each frequency f1 or f2 when they were the deviant (d) or standard (s) stimulus, respectively. The SI was separately calculated for each frequency and it is defined as


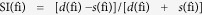


where i = 1 or 2. The positive CSI and SI values indicate neurons respond more strongly to the frequencies when they were deviant compared to when they were standard. To study the contribution of the spontaneous activity on the estimation of the CSI, we calculated again the SSA indices from the evoked activity but with subtracted spontaneous activity bin by bin (evoked activity minus spontaneous activity in spikes/s). The spontaneous activity was estimated within a 50 ms window before each tone presentation in the oddball paradigm (50 ms x 400 trials = 20 s sample window) as previously used^23^.

The best frequency (BF, frequency evoking the most spikes at high intensity levels), characteristic frequency (CF, frequency producing a response at the lowest intensity level) and the threshold were identified. To estimate the temporal response, the BF (100 ms with a 5 ms rise/fall time) was played 500 times at 4 Hz and the regularity of firing was measured by calculating the coefficient of variation (CV, *i.e*., the ratio of the standard deviation to the mean of the interspike intervals) as a function of time over the neuronal response (binwidth=500 μs). A regular response was defined as CV < 0.5 as used by Rees *et al.*, 1997. Also, the monotonicity index (MI, *i.e*., the ratio of the spike count at 80 dB SPL to the maximum spike count) that refers to the degree of reduced spiking at higher intensities was calculated from the FRA measure^59^. Monotonic responses were those with a MI >0.75. Finally, we measured the sharpness of the frequency response area by calculating the bandwidth and Q-values at 10 and 30 dB SPL above the threshold as in our previous work^9^,^21^. The bandwidth at *n* dB expresses the difference in kHz between the lower and upper frequencies of the FRA (BW*n* = FU−FL). The Q-value was calculated as the characteristic frequency divided by the bandwidth at *n* dB above threshold (Q*n* = CF/BW). Results are reported as median ± standard deviation (s.d.) and represented in plots as median ± standard error (s.e.m.).

### Histology and analysis of retrograde labeling

All histological procedures on the fixed brains from the experimental animals were carried out at the University of Connecticut Health Center. Brains were embedded in a gelatin/albumin matrix and transverse, frozen sections were cut at 40 μm thickness through the brainstem. Two series of sections at 240 μm intervals were stained for Nissl substance with cresyl violet and thionin (1:1). Another set of sections at 120 μm intervals was used for FG immunohistochemistry.

Immunohistochemical methods were used to reveal the neurons retrogradely labeled by FG. After 20 min in 0.5% H_2_O_2_ to remove endogenous peroxidase, sections were rinsed in 0.05 M phosphate buffered saline (PBS) and exposed to 10% goat serum with 0.5% Triton X-100 in PBS for 2 hr. Sections were then incubated in anti-FG antibody made in rabbit (Fluorochrome LLC, 1:50000) in the blocker at 4 °C overnight. Following PBS rinses, tissue was exposed to a biotinylated anti-rabbit secondary antibody made in goat (Vector Laboratories; 1:800) in 10% goat serum blocker for 2 hours at 4 °C. The biotinylated secondary antibody was visualized with the ABC-peroxidase method (ABC Elite, Vector Laboratories) performed 2 hr to overnight; and this was followed by preincubation in 0.05% diaminobenzidine with 0.02% cobalt chloride and 0.02% nickel ammonium sulfate (15 min) and the same solution with 0.005% H_2_O_2_ (15 min or less). Sections were dried onto subbed slides and coverslipped with Permount (Fisher Scientific, Pittsburgh, PA).

The injection site (Fig. 4) and retrograde labeling were localized relative to the cytoarchitecture of defined regions in the brainstem, midbrain, and cortex. A mosaic brightfield image was obtained for the complete IC section at the center of the FG injection on a Zeiss Axiovert 200M microscope using AxioVision Rel. 4.8 (Carl Zeiss Imaging Solutions) with a ×10/0.45 NA Planapo lens. An image processing routine was used to visualize the area of the injection site. The image was blurred over a 20 pixel radius, and the background and areas without labeling were removed. The remaining signal representing the FG labeling at the injection site was subdivided into thirds to produce three zones representing heavy, intermediate, and light FG labeling in the injection site. Contours representing those zones were superimposed on the original image and on plots of the relevant sections.

After the examination of all processed sections, Neurolucida (MBF Bioscience Inc., Williston, VT) was used to draw the section outlines and location of the injection sites and the retrogradely labeled neurons. Nissl-stained sections adjacent to the plotted sections were used to draw the cytoarchitectonic boundaries of IC subdivisions, the left auditory cortex, and the nuclei of the lower auditory brainstem. Nissl cytoarchitecture and plots were combined to illustrate the highest density labeling of each structure containing labeled neurons.

## Author Contributions

M.S.M. and D.L.O. designed the experiments, Y.A.A. performed the electrophysiological experiments and data analysis, A.U., K.D., D.B. and D.L.O. performed the histological experiments and data plotting, Y.A.A., M.S.M. and D.L.O. wrote the manuscript.

## Additional Information

**How to cite this article**: Ayala, Y. A. *et al.* Differences in the strength of cortical and brainstem inputs to SSA and non-SSA neurons in the inferior colliculus. *Sci. Rep.* **5**, 10383; doi: 10.1038/srep10383 (2015).

## References

[b1] Ulanovsky N., Las L. & Nelken I. Processing of low-probability sounds by cortical neurons. Nat Neurosci. 6, 391–398 (2003). 12652303 10.1038/nn1032

[b2] Gutfreund Y. Stimulus-specific adaptation, habituation and change detection in the gaze control system. Biol. Cybern. 106, 657–668 (2012). 22711216 10.1007/s00422-012-0497-3

[b3] Nelken I. & Ulanovsky N. Mismatch negativity and simulus-specific adaptation in animal models. J. Psychophysiol. 21, 214 –223 (2007).

[b4] Escera C. & Malmierca M. S. The auditory novelty system: an attempt to integrate human and animal research. Psychophysiology 51, 111–123 (2014). 24423134 10.1111/psyp.12156

[b5] Malmierca M. S., Sanchez-Vives M. V., Escera C. & Bendixen A. Neuronal adaptation, novelty detection and regularity encoding in audition. Front Syst. Neurosci. 8, 111 (2014). 25009474 10.3389/fnsys.2014.00111 PMC4068197

[b6] Anderson L. A., Christianson G. B. & Linden J. F. Stimulus-specific adaptation occurs in the auditory thalamus. J Neurosci 29, 7359–7363 (2009). 19494157 10.1523/JNEUROSCI.0793-09.2009 PMC6666468

[b7] Antunes F. M., Nelken I., Covey E. & Malmierca M. S. Stimulus-specific adaptation in the auditory thalamus of the anesthetized rat. PLoS One 5, e14071 (2010). 21124913 10.1371/journal.pone.0014071 PMC2988819

[b8] Pérez-González D., Malmierca M. S. & Covey E. Novelty detector neurons in the mammalian auditory midbrain. Eur. J. Neurosci. 22, 2879–2885 (2005). 16324123 10.1111/j.1460-9568.2005.04472.x

[b9] Malmierca M. S., Cristaudo S., Perez-Gonzalez D. & Covey E. Stimulus-specific adaptation in the inferior colliculus of the anesthetized rat. J. Neurosci. 29, 5483–5493 (2009). 19403816 10.1523/JNEUROSCI.4153-08.2009 PMC2715893

[b10] Ayala Y. A. & Malmierca M. S. Stimulus-specific adaptation and deviance detection in the inferior colliculus. Front Neural Circuits 6, 89 (2013). 23335883 10.3389/fncir.2012.00089 PMC3547232

[b11] Malmierca M. S. Auditory system. in The rat nervous system (ed. Paxinos G. ) (Academic Press, Amsterdam, 2015).

[b12] Morest D. K. The lateral tegmental system of the midbrain and the medial geniculate body: Study with Golgi and Nauta methods in cat. J. Anat. 99, 611–634 (1965). 17105147 PMC1270700

[b13] Lee C. C. & Sherman S. M. On the classification of pathways in the auditory midbrain, thalamus, and cortex. Hear Res. 276, 79–87 (2011). 21184817 10.1016/j.heares.2010.12.012 PMC3108009

[b14] Winer J. A. Three systems of descending projections to the inferior colliculus. in The Inferior Colliculus (ed. Winer J. A. & Schreiner C. E. ) 231–247 (Springer, New York, 2005).

[b15] Malmierca M. S. & Ryugo D. K. Descending connections of auditory cortex to the midbrain and brainstem. in The Auditory Cortex. (ed. Winer J. A. & Schreiner C. E. ) 189–208 (Springer, New York, 2011).

[b16] Duque D., Perez-Gonzalez D., Ayala Y. A., Palmer A. R. & Malmierca M. S. Topographic distribution, frequency, and intensity dependence of stimulus-specific adaptation in the inferior colliculus of the rat. J. Neurosci. 32, 17762–17774 (2012). 23223296 10.1523/JNEUROSCI.3190-12.2012 PMC6621662

[b17] Ayala Y. A., Perez-Gonzalez D., Duque D., Nelken I. & Malmierca M. S. Frequency discrimination and stimulus deviance in the inferior colliculus and cochlear nucleus. Front Neural Circuits 6, 119 (2013). 23335885 10.3389/fncir.2012.00119 PMC3544151

[b18] Lumani A. & Zhang H. Responses of neurons in the rat's dorsal cortex of the inferior colliculus to monaural tone bursts. Brain Res. 1351, 115–129 (2010). 20615398 10.1016/j.brainres.2010.06.066

[b19] Loftus W. C., Malmierca M. S., Bishop D. C. & Oliver D. L. The cytoarchitecture of the inferior colliculus revisited: a common organization of the lateral cortex in rat and cat. Neuroscience 154, 196–205 (2008). 18313229 10.1016/j.neuroscience.2008.01.019 PMC2562950

[b20] Palmer A. R., Shackleton T. M., Sumner C. J., Zobay O. & Rees A. Classification of frequency response areas in the inferior colliculus reveals continua not discrete classes. J. Physiol. 591, 4003–4025 (2013). 23753527 10.1113/jphysiol.2013.255943 PMC3764642

[b21] Hernandez O., Espinosa N., Perez-Gonzalez D. & Malmierca M. S. The inferior colliculus of the rat: a quantitative analysis of monaural frequency response areas. Neuroscience 132, 203–217 (2005). 15780479 10.1016/j.neuroscience.2005.01.001

[b22] Heffner H. E., Heffner R. S., Contos C. & Ott T. Audiogram of the hooded Norway rat. Hear Res. 73, 244–247 (1994). 8188553 10.1016/0378-5955(94)90240-2

[b23] Duque D. & Malmierca M. S. Stimulus-specific adaptation in the inferior colliculus of the mouse: anesthesia and spontaneous activity effects. Brain Struct. Funct. (2014). 10.1007/s00429-014-0862-1 25115620

[b24] Perez-Gonzalez D., Hernandez O., Covey E. & Malmierca M. S. GABA(A)-Mediated Inhibition Modulates Stimulus-Specific Adaptation in the Inferior Colliculus. PLoS One 7, e34297 (2012). 22479591 10.1371/journal.pone.0034297 PMC3315508

[b25] Harms L., *et al.* Mismatch Negativity (MMN) in Freely-Moving Rats with Several Experimental Controls. PLoS One 9, e110892 (2014). 25333698 10.1371/journal.pone.0110892 PMC4205004

[b26] Kudo M. & Niimi K. Ascending projections of the inferior colliculus in the cat: an autoradiographic study. J. Comp. Neurol. 191, 545–556 (1980). 7419733 10.1002/cne.901910403

[b27] Oliver D. L., Kuwada S., Yin T. C., Haberly L. B. & Henkel C. K. Dendritic and axonal morphology of HRP-injected neurons in the inferior colliculus of the cat. J. Comp. Neurol. 303, 75–100 (1991). 2005240 10.1002/cne.903030108

[b28] Saldana E. & Merchan M. A. Intrinsic and commissural connections of the rat inferior colliculus. J. Comp. Neurol. 319, 417–437 (1992). 1376335 10.1002/cne.903190308

[b29] Malmierca M. S., Seip K. L. & Osen K. K. Morphological classification and identification of neurons in the inferior colliculus: a multivariate analysis. Anat Embryol (Berl) 191, 343–350 (1995). 7645760 10.1007/BF00534687

[b30] Oliver D. L. Dorsal cochlear nucleus projections to the inferior colliculus in the cat: a light and electron microscopic study. J. Comp. Neurol. 224, 155–172 (1984). 19180810 10.1002/cne.902240202

[b31] Malmierca M. S., Saint Marie R. L., Merchan M. A. & Oliver D. L. Laminar inputs from dorsal cochlear nucleus and ventral cochlear nucleus to the central nucleus of the inferior colliculus: two patterns of convergence. Neuroscience 136, 883–894 (2005). 16344158 10.1016/j.neuroscience.2005.04.040

[b32] Coleman J. R. & Clerici W. J. Sources of projections to subdivisions of the inferior colliculus in the rat. J. Comp. Neurol. 262, 215–226 (1987). 3624552 10.1002/cne.902620204

[b33] Oliver D. L., Ostapoff E. M. & Beckius G. E. Direct innervation of identified tectothalamic neurons in the inferior colliculus by axons from the cochlear nucleus. Neuroscience 93, 643–658 (1999). 10465448 10.1016/s0306-4522(99)00143-8

[b34] Oliver D. L., Beckius G. E. & Shneiderman A. Axonal projections from the lateral and medial superior olive to the inferior colliculus of the cat: a study using electron microscopic autoradiography. J. Comp. Neurol. 360, 17–32 (1995). 7499562 10.1002/cne.903600103

[b35] Winer J. A., Larue D. T. & Pollak G. D. GABA and glycine in the central auditory system of the mustache bat: structural substrates for inhibitory neuronal organization. J. Comp. Neurol. 355, 317–353 (1995). 7636017 10.1002/cne.903550302

[b36] Gonzalez-Hernandez T., Mantolan-Sarmiento B., Gonzalez-Gonzalez B. & Perez-Gonzalez H. Sources of GABAergic input to the inferior colliculus of the rat. J. Comp. Neurol. 372, 309–326 (1996). 8863133 10.1002/(SICI)1096-9861(19960819)372:2<309::AID-CNE11>3.0.CO;2-E

[b37] Malmierca M. S., Leergaard T. B., Bajo V. M., Bjaalie J. G. & Merchan M. A. Anatomic evidence of a three-dimensional mosaic pattern of tonotopic organization in the ventral complex of the lateral lemniscus in cat. J. Neurosci. 18, 10603–10618 (1998). 9852596 10.1523/JNEUROSCI.18-24-10603.1998 PMC6793352

[b38] Malmierca M. S., Hernandez O., Antunes F. M. & Rees A. Divergent and point-to-point connections in the commissural pathway between the inferior colliculi. J. Comp. Neurol. 514, 226–239 (2009). 19296464 10.1002/cne.21997 PMC2771101

[b39] Oliver D. L. Ascending efferent projections of the superior olivary complex. Microsc. Res. Tech. 51, 355–363 (2000). 11071719 10.1002/1097-0029(20001115)51:4<355::AID-JEMT5>3.0.CO;2-J

[b40] Ito T. & Oliver D. L. Origins of Glutamatergic Terminals in the Inferior Colliculus Identified by Retrograde Transport and Expression of VGLUT1 and VGLUT2 Genes. Front Neuroanat. 4, 135 (2010). 21048892 10.3389/fnana.2010.00135 PMC2967334

[b41] Loftus W. C., Bishop D. C. & Oliver D. L. Differential patterns of inputs create functional zones in central nucleus of inferior colliculus. J. Neurosci. 30, 13396–13408 (2010). 20926666 10.1523/JNEUROSCI.0338-10.2010 PMC2966845

[b42] Hu B. Functional organization of lemniscal and nonlemniscal auditory thalamus. Exp. Brain Res. 153, 543–549 (2003). 12937877 10.1007/s00221-003-1611-5

[b43] Rees A., Sarbaz A., Malmierca M. S. & Le Beau F. E. Regularity of firing of neurons in the inferior colliculus. J. Neurophysiol. 77, 2945–2965 (1997). 9212248 10.1152/jn.1997.77.6.2945

[b44] Malmierca M. S., *et al.* A discontinuous tonotopic organization in the inferior colliculus of the rat. J. Neurosci. 28, 4767–4776 (2008). 18448653 10.1523/JNEUROSCI.0238-08.2008 PMC2440588

[b45] Anderson L. A. & Malmierca M. S. The effect of auditory cortex deactivation on stimulus-specific adaptation in the inferior colliculus of the rat. Eur. J. Neurosci. 37, 52–62 (2012). 23121128 10.1111/ejn.12018

[b46] Schofield B. R. Projections from auditory cortex to midbrain cholinergic neurons that project to the inferior colliculus. Neuroscience 166, 231–240 (2010). 20005923 10.1016/j.neuroscience.2009.12.008 PMC2814949

[b47] Friston K. A theory of cortical responses. Philos. Trans. R Soc. Lond B Biol. Sci. 360, 815–836 (2005). 15937014 10.1098/rstb.2005.1622 PMC1569488

[b48] Kiebel S. J., Daunizeau J. & Friston K. J. Perception and hierarchical dynamics. Front Neuroinform 3, 20 (2009). 19649171 10.3389/neuro.11.020.2009 PMC2718783

[b49] Bendixen A., SanMiguel I. & Schroger E. Early electrophysiological indicators for predictive processing in audition: a review. Int. J. Psychophysiol. 83, 120–131 (2012). 21867734 10.1016/j.ijpsycho.2011.08.003

[b50] Bendixen A. Predictability effects in auditory scene analysis: a review. Front Neurosci. 8, 60 (2014). 24744695 10.3389/fnins.2014.00060 PMC3978260

[b51] Cauller L. Layer I of primary sensory neocortex: where top-down converges upon bottom-up. Behav. Brain Res. 71, 163–170 (1995). 8747184 10.1016/0166-4328(95)00032-1

[b52] Bajo V. M. & King A. J. Cortical modulation of auditory processing in the midbrain. Front Neural Circuits 6, 114 (2013). 23316140 10.3389/fncir.2012.00114 PMC3539853

[b53] Slater B. J., Willis A. M. & Llano D. A. Evidence for layer-specific differences in auditory corticocollicular neurons. Neuroscience 229, 144–154 (2013). 23137545 10.1016/j.neuroscience.2012.10.053 PMC3534900

[b54] Stebbings K. A., Lesicko A. M. & Llano D. A. The auditory corticocollicular system: molecular and circuit-level considerations. Hear Res. 314, 51–59 (2014). 24911237 10.1016/j.heares.2014.05.004 PMC4140214

[b55] Malmierca M. S., Merchan M. A., Henkel C. K. & Oliver D. L. Direct projections from cochlear nuclear complex to auditory thalamus in the rat. J. Neurosci. 22, 10891–10897 (2002). 12486183 10.1523/JNEUROSCI.22-24-10891.2002 PMC6758437

[b56] Näätänen R. & Winkler I. The concept of auditory stimulus representation in cognitive neuroscience. Psychol. Bull 125, 826–859 (1999). 10589304 10.1037/0033-2909.125.6.826

[b57] Malmierca M. S., Hernandez O. & Rees A. Intercollicular commissural projections modulate neuronal responses in the inferior colliculus. Eur. J. Neurosci. 21, 2701–2710 (2005). 15926918 10.1111/j.1460-9568.2005.04103.x

[b58] Näätänen R. Attention and brain function. Hillsdale, NJ: Lawrence Erlbaum. (1992).

[b59] Watkins P. V. & Barbour D. L. Level-tuned neurons in primary auditory cortex adapt differently to loud versus soft sounds. Cereb Cortex 21, 178–190 (2011). 20457692 10.1093/cercor/bhq079 PMC3000570

